# Dextro-Quinine in Pertussis

**Published:** 1879-11-20

**Authors:** J. L. Coombs

**Affiliations:** Grass Valley, Cal.


### *DEXTRO-QUININE IN PERTUSSIS.*

BY J. L. COOMBS, M.D., GRASS VALLEY, CAL.

In these mountainous regions, in which Grass Valley is situated, we have no endemic intermittent fever, but nearly all cases of fever are remittent, and of the class in which quinine is believed to be indicated.

*Case* 1.—Frank Roberts, male, æt. three and a half years. Debility, emaciation, broncho-pneumonia, with fever remittent, and worm-symptoms prominent, ascarides seen.

With other treatment, as indicated, quinia sulph., P. & W., was used during remission of fever, and after annoying cerebral symptoms (quinism) were produced, a convalescence seemed established, and one dose of three grains was ordered every morning. I ceased attendance.

Ten days later I was again called; child had same general febrile action, but all respiratory symptoms were absent, save an irritative cough during exacerbation of fever. This patient was in the first stage of Pertussis when I first saw him. After a purgative dose of calomel and scammony, combined with santonine, I gave during remission and exacerbation of fever alike, four grains of dextro-quinine, every three hours until twenty grains had been used, when the tongue had become natural in appearance, save slight redness, which disappeared next day; and since then, two months, recovery progressed, and he is well.

*Case* 2.—Bessie Hoyne, æt. three years and eight months, girl. Remittent fever, of one week's duration, under other treatment. Worm-symptoms prominent.

Used same purgative dose as in case 1st. Gave aconite and belladonna alternately during exacerbation, at first, but afterward used gelsemium and quinia sulph. during remission.

In this case I was really disappointed in the lack of influence from quinine, and although eight grains were used for a dose, no perceptibly unpleasant, nor any other effect seemed induced, save it may have been drowsiness increased, and not of the kind produced by gelsemium, and which had preceded my treatment. I bethought me of the "dextro," and gave four grains every three hours persistently, and in thirty-six hours I ceased all medicine, save one grain of dextro-quinine each morning for a week more.

Emaciation and extreme debility, very rapidly increasing, was the marked characteristic of these two cases. Both had been preceded by whooping cough, for which they had received no treatment until febrile symptoms set in. The cough was wonderfully convulsive and violent in each case.

No so-called cough remedies were prescribed, yet so soon as the effect of the dextro-quinine was had, *all cough ceased at once*, which I consider remarkable. Quinia sulphate and mur. ammon. have been recommended for pertussis for many years, but, although I have used it, with some success, nothing like the results ever followed which I recognize as the immediate results in these two and another case, (in which I used it alone, but in which no febrile symptoms were observed), of the use of dextro-quinine. Hereafter I shall use it in pertussis as a remedy, if not contra-indicated, with a hope that it shall prove as specific as the disease itself.

In the June number of Virginia Medical Monthly, page 221, in a short article upon the oxytocic properties of quinine, where I could not give even half grain doses without reproducing uterine hemorrhage, I have just recently used four grain doses of the dextro-quinine with fine tonic effect, and the hemorrhagic discharge seems uninfluenced, although no combination was made with any other remedy. Iron still cannot be borne in the smallest dose, or in any form—dyalised hypodermically, or otherwise. Quinine has the same influences now as it did twenty-three years ago upon the patient.

The writer has always been peculiarly subject to tinnitus aurium after even a fractional dose of sulph. quinine, and even one-sixth gr. has induced the unpleasant sensation.

Yesterday, and the day previously, I took purposely, two doses of eight grs. each, two hours apart, mixed in water, and aside from a warming pleasant sensation in the stomach, and general increased feeling of organic comfort, nothing followed.

---

**Pruritus Ani.**—In simple pruritus ani I have had very excellent results from good glycerole of tar. It must be good, however, and not made of coal tar or some patent wagon grease. If there is hypertrophy of the skin and mucous membrane I like the stramonium ointment made with mutton of tallow and the fresh herb. If there is eczema, as I suppose there is in this case, I should use the brown citrine ointment. If it irritates, rub it up with from one to three parts of simple cerate.—*Eclectic Medical Journal.*

